# Educational

**Published:** 1889-03

**Authors:** 


### Educational.

Below are given some questions from the syllabus of Prof. C. L. Ford, M.D., D.D.S., used in his course of instruction of the dental students in the Dental College of the University of Michigan. They illustrate very clearly the incomparable method of questioning used in the course of instruction by this world-renown teacher of anatomy and physiology. These questions are so tersely and clearly put that the student of former years will find a pleasure in running over them, and a renewal of the subject to which they refer; and in such a manner as would hardly be attained by days of reading on the subject.

These questions are given not so much for this purpose, however, as to illustrate the method employed by this devoted teacher.

QUESTIONS ON STRUCTURE OF TEETH.

1. Name all the tissues found in a human tooth.
2. What part of a tooth does each of these form ?
3. What is the hardest substance in the human body ?
4. What structural elements are found in enamel ?
5. How are the enamel prisms or rods arranged ?
6. Why are they perpendicular on the surface of crown ?
7. How do they differ on the end and side of teeth ?
8. How large are the enamel prisms ; how shaped ?
9. Are they of uniform size in all parts ?
10. In what way are these rods so firmly united ?
11. What proportion of animal and mineral material ?
12. What and where are the transverse striae of retzius ?
13. Does a tooth possess power to repair enamel ?
14. Does the enamel possess ordinary sensibility ?
15. In what way is the enamel united to the dentine ?
16. In development which tissue is formed first ?
17. What tissue of teeth is least in quantity ?
18. What part of a tooth does this substance form ?
19. By what different names is this tissue known ?
20. What other tissue of the body does it resemble ?
21. State in what respect the resemblance consists.
22. What is named as present in normal cement ?
23. What is sometimes found when increased in quantity ?
24. By what name is any increase in cement known ?
25. Is healthy cement endowed with much sensibility ?
26. What tissue makes most of the tooth substance ?
27. Is the dentine everywhere covered ; if so, how ?

28. What part of a tooth is called its ivory? Why?
29. What proportion of animal and mineral material?
30. What is meant by dentine cartilage? How obtained?
31. What is meant by the matrix of a tooth?
32. What is meant by the intertubular substance?
33. What is the nature or character of this substance?
34. What canals or channels are seen in dentine?
35. Describe their commencement and termination.
36. What is their direction through the dentine?
37. How large are these channels in the dentine?
38. Are they equally abundant in all parts of it?
39. In what manner do they branch and anastomose?
40. By what names are these channels known?
41. Do the dental tubuli join canaliculi or cement?
42. Do they ever communicate with spaces in enamel?
43. What immediately surrounds the dental tubuli?
44. What and where is the sheath of Neumann?
45. How are the dental tubuli occupied in life?
46. What is the source of such substance or fibrils?
47. Why do they receive the name usually given?
48. What is ment by the dental sheath, and where?
49. Describe the character and properties of this sheath?
50. What tissue of the teeth has the greatest sensibility?
51. Where is the sensitiveness of dentine greatest?
52. Is dentine sensitive where the pulp is destroyed?
53. What is meant by contour lines? Where seen?
54. What and where in tooth, is the granular layer?
55. What and where are the interglobular spaces?
56. Are these appearances found in all teeth?
57. Are they the result of imperfect development?
58. What is the tooth pulp, and where situated?
59. Of what is this substance composed? Its color?
60. How do nerves and blood-vessels reach the pulp?
61. What other constituents assist to form the pulp?
62. Of what structure is the pulp the remains?
63. What is meant by the membrana eboris?
64. Where are found the odontoblast cells?

65. Describe these cells and their processes.
66. Where and what are Tomes fibres or tooth fibrils?
67. With what are they connected in the pulp?
68. What purpose do they serve in the teeth?
69. Are Tomes fibers to be considered as nerves?
70. What is Nasmyth's membrane, and where found?
71. State concisely what you learn concerning it.

